# Evolution of Microstructure and Mechanical Properties of Ti-6Al-4V Alloy under Heat Treatment and Multi-Axial Forging

**DOI:** 10.3390/ma17051060

**Published:** 2024-02-25

**Authors:** Sijie Du, Yang Song, Yiting He, Chunhua Wei, Rongyou Chen, Shubo Guo, Wei Liang, Shengyuan Lei, Xiaohong Liu

**Affiliations:** 1 School of Resources, Environment and Materials, Guangxi University, Nanning 530004, China; dsj15577168431@163.com (S.D.); 414067874@139.com (Y.S.); heyiting87@sina.com (Y.H.); 2115391005@st.gxu.edu.cn (R.C.); shuboguo@163.com (S.G.); 2215391057@st.gxu.edu.cn (W.L.); lei-sy@hotmail.com (S.L.); liuxiaohong@gxu.edu.cn (X.L.); 2 State Key Laboratory of Featured Metal Materials and Life-Cycle Safety for Composite Structures, Guangxi University, Nanning 530004, China

**Keywords:** Ti-6Al-4V alloys, heat treatment, multi-axial forging, ultrafine-grain, bimodal structure, mechanical properties

## Abstract

The mechanical properties of various Ti-6Al-4V alloys are influenced by their respective microstructures. This study generated an ultrafine-grain (UFG) Ti-6Al-4V alloy featuring bimodal grain distribution characteristics achieved through initial heat treatment, multi-axial forging (MF), and annealing. The study also extensively examined the evolution process of the alloy’s microstructure. By subjecting the materials to heat treatments at 900 °C with air cooling and 950 °C with air cooling, both materials were found to be consisted of primary α (α_p_) and transformed β (α_s_+β) regions with different proportions. Following MF, the sample treated at 900 °C displays a microstructure featuring UFGs of α+β surrounding larger micron-sized α_p_ grains. On the other hand, the sample treated at 950 °C displays a microstructure distinguished by twisted α_s_ lamellar and fragmented β grains surrounding larger micron-sized α_p_ grains. Following annealing, no significant grain growth was observed in the sample. The geometrically necessary dislocations (GNDs) within the UFGs were eliminated, though some GNDs persisted within the α_p_ grains. The samples undergoing the 900 °C heat treatment, MF, and subsequent annealing exhibited elevated strength (1280 MPa) and total elongation (10.7%). This investigation introduces a novel method for designing the microstructure of the Ti-6Al-4V alloy to achieve superior performance.

## 1. Introduction

The Ti-6Al-4V alloy is widely acknowledged for its outstanding properties, encompassing high specific strength, exceptional fracture toughness, resistance to fatigue, corrosion, and heat, as well as resilience against low-temperature embrittlement. Additionally, it possesses a low thermal expansion coefficient [1]. As a result, the Ti-6Al-4V alloy has been widely employed in diverse industries, including aerospace, automotive, biomedical implants, gas turbines, and chemical engineering [2,3]. Nevertheless, due to advancements in industrial demands, the conventional Ti-6Al-4V alloy no longer fulfills the requirements of specific specialized applications.

Reducing the grain size is a widely acknowledged method for enhancing strength, hardness, resistance to high cyclic fatigue, and superplasticity. Ultrafine-grained (UFG) or nano-crystalline (NC) microstructures can be achieved within metal materials through severe plastic deformation (SPD) processing. In the last two decades, there has been a notable emphasis on researching the production of UFG materials through SPD. Titanium alloys, such as Ti-6Al-4V, can be effectively processed using a range of SPD techniques, including Multi-axial Forging (MF) [4], High-Pressure Torsion (HPT) [5], Equal Channel Angular Pressing (ECAP) [6,7], Constrained Groove Pressing (CGP) [8,9], and Accumulative Roll Bonding (ARB) [10]. Employing diverse heat treatment processes on the Ti-6Al-4V alloy can yield a range of microstructures, including the fully equiaxed structure [11], widmanstätten structure [12], bimodal structure [13], martensitic structure [14], lamellar structure [15], etc. Distinct initial microstructures lead to varied microstructures after SPD. Bhardwaj [16] subjected a Ti-6Al-4V alloy sample with an initial equiaxed structure to CGP at 550 °C, producing a consistently equiaxed structure with an average grain size of 13.9 μm. This process resulted in an ultimate tensile strength (UTS) of 1118.3 MPa and a total elongation (TE) of 4.9%. Chao [17] performed asymmetric rolling (AsR) processing on Ti-6Al-4V alloy samples initially characterized by a martensite microstructure at 800 °C. This led to the formation of a UFG microstructure with an average grain size of 110 nm and a distinct texture along the rolling direction. Its UTS achieved 1365 MPa, and TE exhibited a 22.6% increase compared to an AR plate in the rolling direction. Zherbtsov et al. [18] utilized MF on Ti-6Al-4V alloy samples possessing an initial martensite microstructure within the temperature range of 700 °C~450 °C. This process yielded an equiaxed UFG microstructure with an average grain size of 150 nm. The UTS achieved 1400 MPa, and TE was measured at 6.8%. Zhang [15] conducted multi-directional isothermal forging (MDIF) on Ti-6Al-4V alloy samples that possessed an initial widmanstätten microstructure within the temperature range of 950 °C~850 °C. This process yielded a consistently equiaxed UFG microstructure at the micrometer level. The UTS reached 921 MPa, and TE was measured at 18.4%. X. Ding et al. [19] controlled the original grain size through different heat treatment temperatures and then carried out a three-roll screw-rolling process and subsequent heat treatment on Ti-6Al-4V alloy bars. Finally, a material with significant differences in microstructure and mechanical properties was obtained, and the results showed that the bimodal microstructure with α grains accounting for about 40% had higher tensile strength when the area reduction rate was close. Hu [20] treated a Ti-6Al-4V alloy sample characterized by a bimodal structure through HPT processing at room temperature, resulting in a nanoscale equiaxed UFG microstructure. After two and four passes of HPT, the UTS and TE reached 827 MPa, 8.0%, and 960 MPa, 0.4%, respectively. The above studies suggest that the exploration of equiaxed UFG material preparation using SPD in the Ti-6Al-4V alloys with various initial microstructures has been relatively comprehensive.

In recent years, there has been a growing interest in enhancing the total elongation of UFG materials. Researchers have turned their attention to exploring the application of a bimodal microstructure in various UFG titanium metal materials. In fact, the concept of bimodal microstructure not only exists in the field of metal materials but also has corresponding applications in many research fields [21,22]. This microstructure involves the creation of a bimodal structure characterized by relatively coarse recrystallized grains embedded in a nanostructured matrix [18]. This structure fundamentally enhances the material’s capability to accommodate dislocations during deformation by introducing a fraction of coarse grains. The plastic deformation of each phase in multiple-phase alloys is interdependent, and various crystal structures and properties exhibit distinct abilities to adapt to strain. Hence, the nanocrystallization mechanism of α+β phase alloy is more intricate [23]. Wu et al. [24] engineered a heterogeneous lamellar structure in Ti through AsR and partial recrystallization, leading to high strength and significant elongation. Xu et al. [25] produced pure titanium with a multimodal grain structure consisting of nanoscale, ultrafine, and coarse grains through room-temperature cold rolling and short-duration annealing. The resulting material exhibited high strength and excellent plasticity. Zherebtsov et al. [18] performed MF on the Ti-6Al-4V alloy characterized by a microstructure consisting of primary global α and lamellar α/β. Ultimately, they achieved a bimodal UFG microstructure with an average grain size of approximately 300 nm and a UFG proportion of 0.4, effectively enhancing the material’s elongation. I.V. Vlasov et al. [26] prepared VT8 alloy with bimodal grain structure using helical rolling and additional air quenching processes, which eliminated the negative impact of long α layers on the material’s impact toughness. Due to the formation of equiaxed α-Ti grains and the increase in grain boundaries, the material’s impact toughness was improved.

In summary, previous studies have indicated that the preparation of a fully UFG Ti-6Al-4V alloy through SPD tends to result in reduced elongation. Conversely, introducing a bimodal UFG microstructure has shown promise in addressing the issue of total elongation. Nevertheless, there is still a need for further research on the microstructure evolution process of the bimodal UFG formed by SPD in the two-phase Ti-6Al-4V alloy. This study modifies the microstructure and composition of the Ti-6Al-4V alloy through heat treatment, achieving a bimodal microstructure in the alloy using MF and subsequent annealing. The study explores the microstructure evolution and mechanical properties of the Ti-6Al-4V alloy with the newly formed bimodal UFG microstructure.

## 2. Materials and Methods

### 2.1. Materials

The material employed in this study is a 10 mm thick annealed plate of Ti-6Al-4V titanium alloy. The measured chemical composition (wt%) of the plate can be found in Table 1. The experimentally determined β/α+β transition temperature (T_β_) is approximately 985 °C.

**Figure 1:**
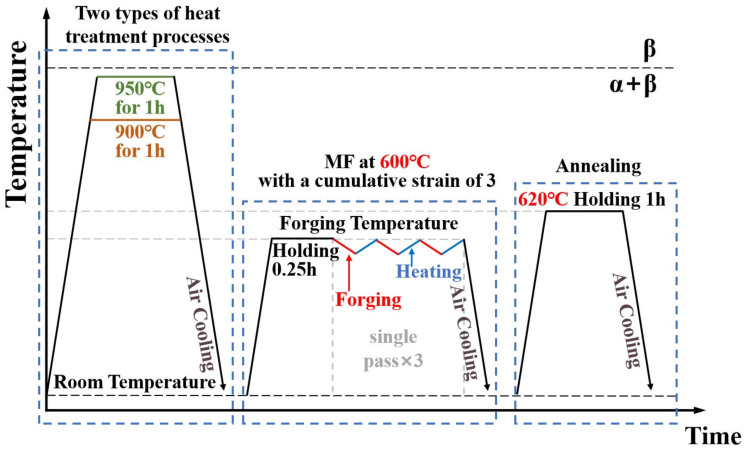
Schematic diagram illustrates the heat treatment, MF, and annealing processes.

Cubic samples measuring 10 × 10 × 10 mm^3^ in length, width, and thickness were obtained from the as-received (AR) plate using wire-cut electrical discharge machining (EDM). The samples underwent two sets of distinct treatments, and the procedures for each route in both processing methods are illustrated in Figure 1. Heat treatment at a temperature slightly lower than T_β_ can result in the coexistence of equiaxed grains and lamellar structures in the alloy. Therefore, heat treatment was chosen at two temperatures: 900 °C and 950 °C. In the initial stage, the samples underwent homogenization for 1 h at 900 °C and 950 °C, respectively, followed by air cooling. They were called the 900T and 950T samples, respectively. Subsequently, the samples underwent forging at 600 °C, with each forging cycle reducing the thickness by 33%. This allows the sample to undergo sufficient plastic deformation without cracking. Three consecutive cycles were performed in the x-y-z multiple axials, constituting one pass. After three passes, the sample with a length, width, and thickness of approximately 10 × 10 × 10 mm^3^.

The alloys subjected to MF were denoted as the 900T-MF and 950T-MF samples, respectively. After MF, the samples underwent annealing at 620 °C, which were designated as the 900T-MF-A and 950T-MF-A samples, respectively.

### 2.2. Characterization of Microstructure

The sample was bisected along its center using EDM. Subsequently, the sample cross-section underwent mechanical polishing before being subjected to observation. They were polished using SiC papers of 400, 1000, 2000, and 3000 grades, followed by diamond lapping to achieve a smooth and micro-scratch-free surface. Following this, chemical etching was carried out using a solution composed of 2 mL HF, 5 mL H_2_O_2,_ and 93 mL H_2_O. Microstructure analysis was conducted using an optical microscope (OM, Motic KPA53MET-BD, Okayama, Japan) and a scanning electron microscope (SEM, HITACHI S-3400N, St. John’s, NL, Canada). Five randomly chosen regions of OM and TEM images were analyzed in the assessment, and the ImageJ (1.8.0) software was employed to calculate the number of grains per unit area. Firstly, the size of each α_p_ grain was counted through OM images, and the area of all α_p_ grains and UFG (α_s_+β) grains were calculated to determine the proportion of α_p_ grains and UFG (α_s_+β) grains in the overall structure. Afterward, the size of UFG (α_s_+β) grains was statistically analyzed using TEM images. Finally, draw a grain statistics chart based on the respective proportions of α_p_ grains and UFG. The dimensions of microstructural features, including grains and lamellar thickness, were estimated using the Line intercept Method.

Adhering to the standard transmission electron microscopy (TEM) sample preparation procedure, the sample underwent mechanical thinning, reducing its thickness to 50~60 µm. Subsequently, a 3 mm circle was extracted from the sample, double-sprayed with the Struers Tenupol-5 double spray instrument, and the thin area was cleaned using the argon ion thinning instrument (Gatan 691) with a voltage of 1.0 keV to achieve a continuous and high-quality thin section. The material’s microstructure was characterized using a TEM, specifically the FEI Tecnai G2 F20, operating at 200 kV. The sample preparation procedure for the transmission Kikuchi diffraction (TKD) test mirrored that of the TEM sample, adhering to the standard process for crystallographic orientation tests. TKD characterization was conducted using the Super Velocity EBSD probe mounted on the JSM 7200F field emission SEM. The test parameters included a step length of 15 nm, a resolution of 400 × 400 pixels, and a voltage of 20 kV. Exposure parameters were optimized to achieve an acquisition rate of approximately 200 frames per second.

The X-ray diffraction (XRD) samples were prepared by electrolytic polishing, with an electrolyte composed of 1:9 perchloric acid and methanol. The voltage and temperature were 20 V and −20 °C, respectively. XRD (BRUKER D8 Discover, Berlin, Germany) tests were performed at the ambient temperature using a diffractometer with a Cu-Kα radiation source (λ = 1.5418 Å). The scanning range and rate for the XRD tests were 30°–80° and 2°/min, respectively.

### 2.3. Performance Testing of Mechanical Properties

In the context of tensile testing, miniaturized samples are meticulously prepared for the alloy under examination, ensuring that the aspect ratio of the sample size adheres to the guidelines outlined in the ASTM E8 standard [27]. Samples with a dog-bone shape, featuring dimensions of 5 mm in gauge length, 1 mm in width, and 0.5 mm in thickness, were crafted using EDM. Tensile tests were subsequently carried out at room temperature utilizing Universal Tensile Machine (Kiatest, KT26.105, Guangzhou, China) with a constant strain rate of 1 mm/min. Three separate tensile tests were conducted for each sample type to ensure data reproducibility. The resulting numerical values for mechanical properties such as UTS and TE and their corresponding standard deviations were determined. Following that, a fracture surface analysis was conducted using SEM. Sample hardness was assessed using a Vickers hardness tester (NVM-1000A, MECANIQUES NAVARRO S.L., Vallada, Spain) with a 2.942 N load and a 15-s dwell time. Each sample underwent nine random hardness tests. The spacing between each indentation is greater than 500 μm.

## 3. Results and Discussion

### 3.1. Evolution of Microstructure during Heat Treatment

**Figure 2:**
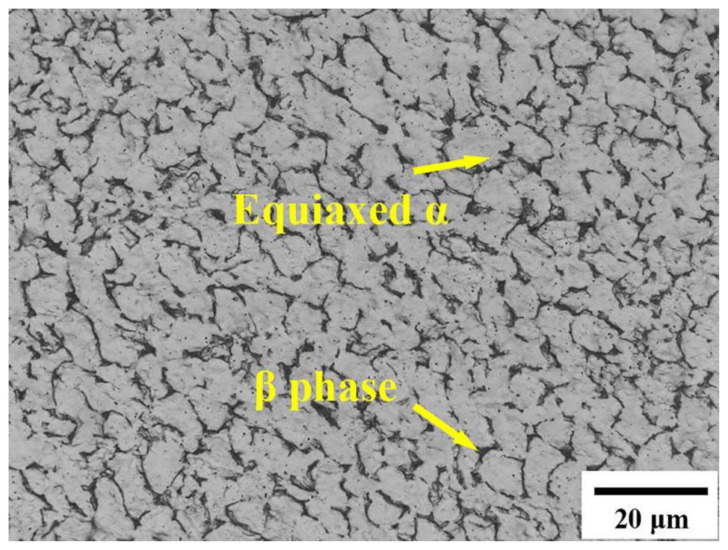
Microstructural characteristics of the AR-Ti-6Al-4V alloy.

Figure 2 displays a representative OM of the as-received AR-Ti-6Al-4V alloy. The microstructure is characterized by equiaxed α grains, and β phases are uniformly distributed along the boundaries of these equiaxed α grains. The volume percentages of V(α) and V(β) are approximately 98.4% and 1.6%, respectively.

**Figure 3:**
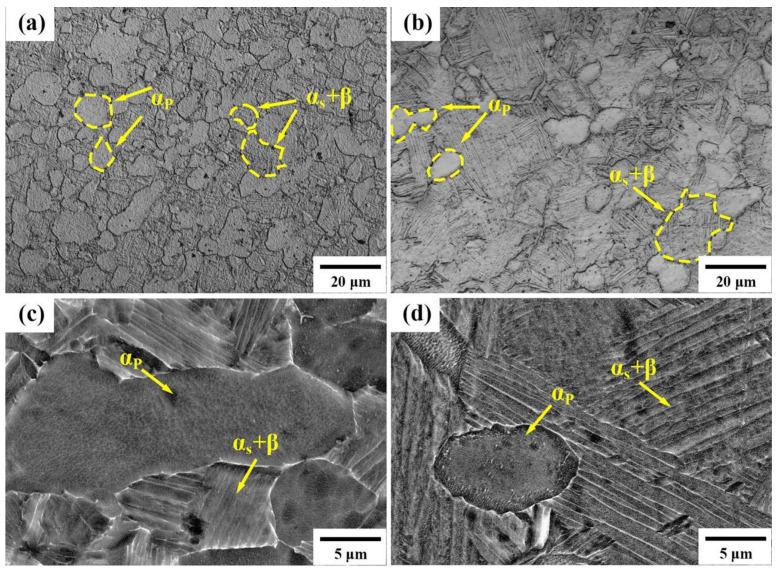
Microstructural analysis of Ti-6Al-4V alloy: (**a**) OM and (**c**) SEM for the 900T sample, and (**b**) OM and (**d**) SEM for the 950T sample.

Based on the OM and SEM images showcased in Figure 3 for the 900T and 950T samples, it is apparent that the microstructure consists of primary α_p_ interspersed with secondary α (α_s_) and β alternating lamellae. This structure is formed during the cooling process of the high-temperature β phase. The analysis revealed that in the 900T sample, the proportion of primary α_p_ phase is 58.7%, while in the 950T sample, it is 12.8%. The average grain size of α_p_ grains in the 900T sample is 16.7 ± 2 µm, and in the 950T sample, it is 5.1 ± 0.8 µm. Additionally, in the 900T sample, the thickness of the secondary α_s_/β lamellar was determined to be 0.4 ± 0.2 µm and 0.08 ± 0.02 µm. In contrast, in the 950T sample, these lamellae exhibited thicknesses of 0.8 ± 0.2 µm and 0.12 ± 0.03 µm.

With the gradual increase in heat treatment temperature approaching T_β_, the primary α_p_ phase started to diminish, enabling the β phase to develop into distinct grains. However, it is important to note that since the temperature did not reach the T_β_, the presence of α_p_ persisted throughout the microstructure. During the subsequent cooling phase, both lamellar secondary α_s_ and β phases precipitated from the high-temperature β phase. Given that the cooling rates for both samples were nearly identical, the morphology of the lamellar α_s_ and β phases formed was essentially the same. The distinction lies in the lower α_p_ content in the 950T sample compared to the 900T sample. This lower content allowed for greater spatial freedom for lamellar structures to expand, leading to longer lengths and thicker widths of the lamellae in the 950T sample. In contrast, the 900T sample, characterized by more pronounced obstacles, produced a transformation microstructure with shorter lengths and narrower lamellar widths. The volume fractions of both α_p_ and α_s_+β in both types of samples, along with the sizes of α_p_ and α_s_+β lamellae, are critical parameters that significantly influence the performance of the alloy [28]. Given that MF will further adjust the organization in the subsequent steps, the primary emphasis of this study does not center on the variations in mechanical properties post-heat treatment. Instead, the primary focus is understanding the differences in the proportion of α_p_ and α_s_+β lamellar phases.

**Figure 4:**
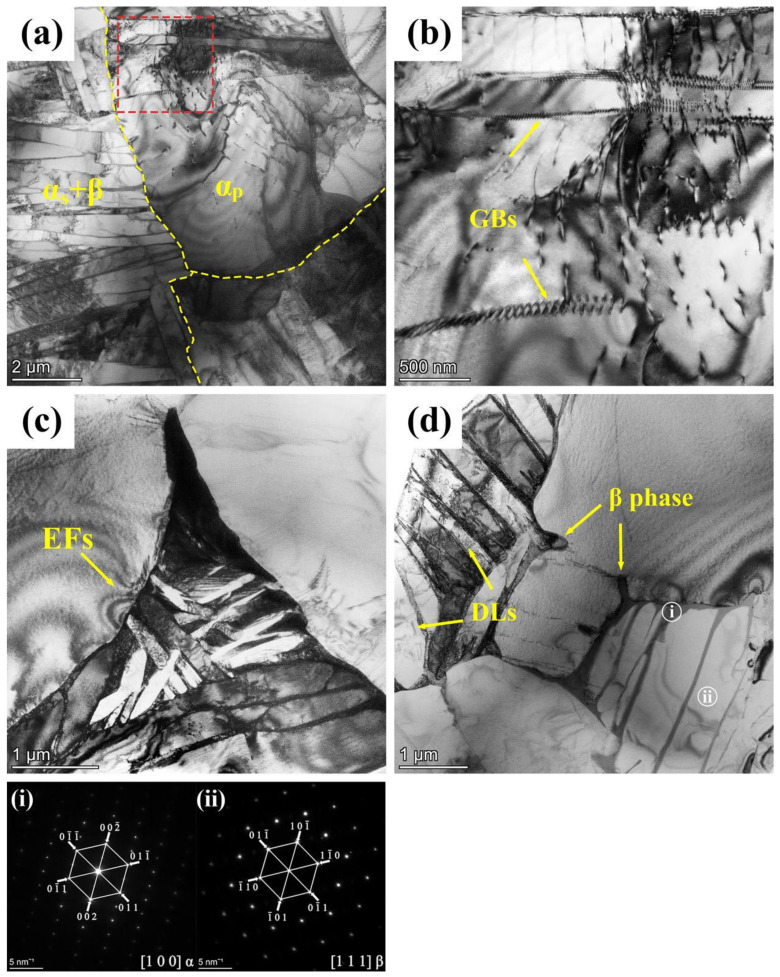
TEM analysis of microstructural evolution in 900T sample: (**a**) Overview of lamellar and equiaxed microstructures; (**b**) Formation of grain boundaries(GBs) in α_p_; (**c**) Growth of lamellar structure at α grain boundaries; (**d**) The process of lamellar structure formation and dislocation lines (DLs) in lamellar structures; (**i**,**ii**) Corresponding SAED patterns for marked areas.

Concerning the disparities between the 900T and 950T samples after heat treatment, the primary distinction lies in the differing proportions of their α_p_ and lamellar structures. This phenomenon is further elucidated through TEM imaging, specifically focusing on the 900T sample in Figure 4. As depicted in Figure 4a, the lamellar are on the left. At the same time, the α_p_ grains are located in the upper right corner. The observation reveals that dislocations are primarily concentrated in the lamellar microstructure region. The lamellar structures of α_s_ and β possess different slip systems and distinct crystal structures, which could account for strain mismatching and dislocations [29]. In Figure 4b, the progression of grain boundary formation within α_p_ grains and the subsequent division of α_p_ grains can be observed, representing the transformation from α_p_ grains to lamellar structures. Additionally, in Figure 4c, a dark triangular region corresponds to the β phase distributed at the boundaries of α_p_ grains. The observation indicates the transition of the β phase into a lamellar structure. Particularly noteworthy are the regions with noticeable variations in equal-thickness fringes (EFs), as indicated by white arrows in Figure 4c. These regions are associated with the growth direction of the lamellar structures, signifying the gradual transformation of α_p_ grains into lamellar structures.

The transformation process of equiaxed grains is also observable. As the new grain boundary forms and divides the grain, the β phase gradually emerges in this region, as indicated by the arrows in Figure 4d. In Figure 4d, the lamellar structure is visible. Selected-area electron diffraction (SAED) was conducted on different regions of the lamellar structure. The results indicate that the white region i corresponds to the α phase, while the gray region ii corresponds to the β phase. An important characteristic of α+β two-phase titanium alloys, in contrast to single-phase β titanium alloys, is the adjustable stability of the β phase resulting from the distribution of elements during heating in the α+β region [30].

### 3.2. Microstructure Evolution during MF

#### 3.2.1. Evolution of α_p_ during MF

**Figure 5:**
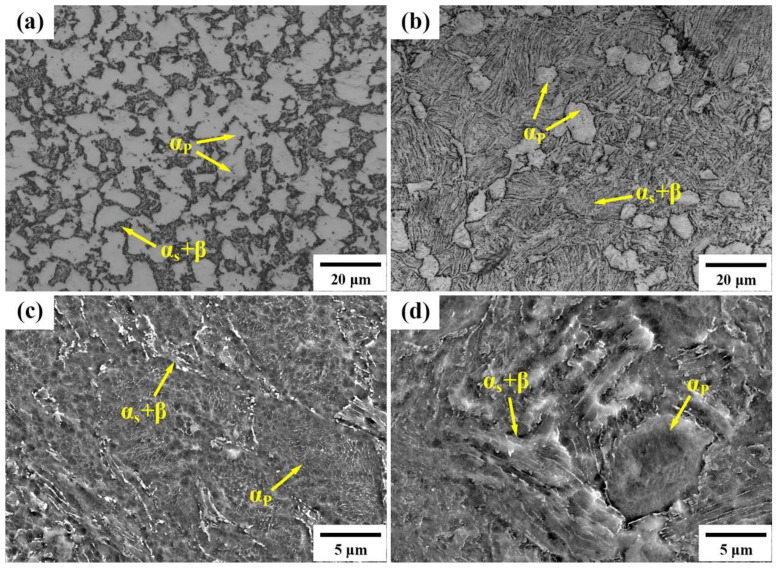
Microstructural analysis of Ti-6Al-4V alloy. The 900T-MF sample: (**a**) OM and (**c**) SEM. The 950T-MF sample: (**b**) OM and (**d**) SEM.

The novelty of the unique process employed in this study primarily resides in controlling the proportion of equiaxed and lamellar structures during the initial heat treatment. Due to differences in the mechanisms of grain fragmentation between equiaxed and lamellar structures, separate discussions are undertaken for equiaxed and lamellar structures. The OM and SEM images in Figure 5 depict the microstructural morphology of the 900T-MF and 950T-MF samples. The OM images presented in Figure 5a,b demonstrate that the α_p_ grains in both the 900T-MF and 950T-MF samples retain their equiaxed morphology after MF. In the SEM images, magnified 4000× in Figure 5c,d, it is noticeable that the α_s_+β lamellar structure in the 900T-MF sample is almost completely broken. Meanwhile, in the 950T-MF sample, the β lamellar are all broken, and the α_s_ lamellar undergoes significant distortion but does not break.

**Figure 6:**
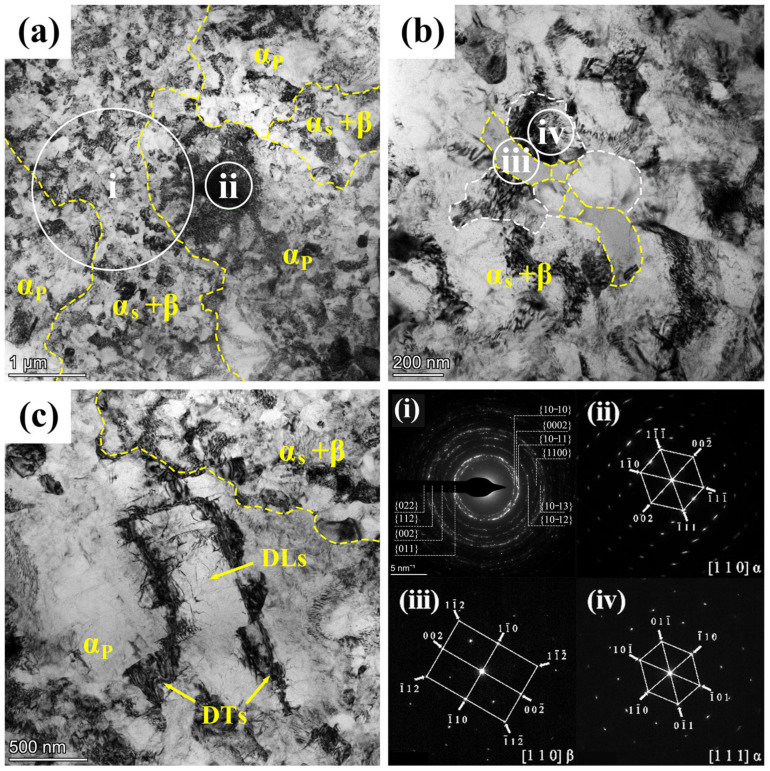
TEM bright field images of the 900T-MF sample. (**a**) Overview of α_p_ and α_s_+β regions microstructures; (**b**) α_s_+β lamellar fracture and globularization process; (**c**) Dislocation tangles (DTs) region in α_p_; (**i**–**iv**) Corresponding SAED patterns for the marked areas.

**Figure 7:**
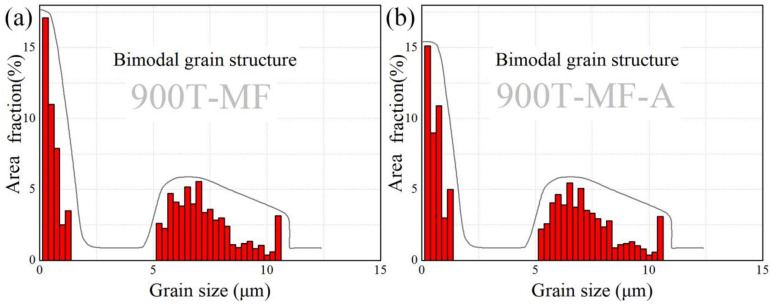
Statistical grain size distribution of (**a**) the 900T-MF sample and (**b**) the 900T-MF-A sample.

Figure 6 depicts the TEM bright field plot of the 900T-MF sample, revealing a typical bimodal grain structure, as illustrated in Figure 6a. This structure consists of coarse grains surrounded by broken finer grains. Through statistical analysis of OM image data from multiple 900T-MF samples, it can be concluded that the grain size of α_p_ grain ranges from 5 to 10 μm. Figure 7 presents the characteristics of bimodal grains. Additionally, region ii in Figure 6a underwent SAED analysis. The diffraction pattern confirms the area as a single crystal structure oriented along the crystallographic axis of [110], identified as the α phase. Notably, the elongated diffraction features implied significant deformation energy accumulation within the grain boundaries, indicating a sub-stable state characterized by high internal stress [31]. Moreover, a tendency to create LABs is observed in areas where dislocation accumulates. Subgrains initiate within α_p_ grains as a result of continuous stress build-up. With increasing deformation, these grains eventually fracture into smaller entities.

Furthermore, numerous dislocation lines (DLs) were observed within the grains, as depicted in Figure 6c. These dislocation lines were randomly distributed throughout the α_p_ phase. Concurrently, high-density dislocation lines are intricately entangled, forming the observed dislocation tangles (DTs) in Figure 6c. It is worth mentioning that a significant number of DTs divide the original coarse grains into two or more elongated regions, as illustrated in Figure 6c. Liu [23] has noted that this structure bears similarity to twinning effects. The DL configuration around the regions with DTs forms a lens-like structure, suggesting a tendency toward twinning. This arrangement implies the presence of stress relief mechanisms within α_p_ beyond the accumulation of dislocations and grain fragmentation.

#### 3.2.2. Evolution of α_s_+β in MF

Analyzing the SEM images in Figure 3, it becomes apparent that the interior of the β-transformed structure exhibits a lamellar morphology, comprising both α and β lamellar. Following MF, notable alterations in the morphology of the β-transformed structure are observed. As depicted in Figure 5, the previously lamellar structure becomes distorted and fragmented. In Figure 6b, the TEM image vividly illustrates the compression and fragmentation of the lamellar structure. Examining this area using SAED indicates that the main part of Region iii is the β phase, whereas region iv comprises the α phase. The average grain size of the broken α_s_+β region in the 900T-MF sample was determined to be 146.9 ± 20 nm by analyzing multiple TEM images. In the process of MF, diverse lattice defects, such as dislocations, twins, and stacking faults, are introduced into the microstructure. The build-up of many dislocations serves as the initiation point for the fracture process [32]. Remarkably, a significant concentration of dislocations is noticeable on one side of the α grains, with the lamellar β phases hindering the mobility of these dislocations. With increasing stress, the lamellar β phases undergo compression and fragmentation, leading to the fracture and generation of fine grains. Wang [33] conducted experiments on a fully lamellar Ti-6Al-4V alloy using both MF and non-monotonic torsion. Their study revealed that the direction of the applied stress notably influences the degree of deformation in the lamellar structure. Deformation in multiple axials can potentially elevate the extent of lamellar structure fragmentation. Nonetheless, in two-phase titanium alloys, our findings indicate that the degree of lamellar structure globularization during MF is intricately linked to the phase proportions of the individual phases.

In contrast to the 900T-MF sample, the 950T-MF sample yields a distinct result due to variations in lamellar structures. Figure 5d illustrates that the β phase transforms its initial lamellar form to scattered points independently distributed between two distorted α lamellae. This suggests that the β lamellar structure is the first to undergo breakage during the deformation process. According to Froes [34], this phenomenon is attributed to the softer nature of the β phase (BCC) compared to the α phase (HCP), causing it to undergo more pronounced deformation and preferential spheroidization when subjected to external forces. Additionally, there were observations of β penetration along the twisted α_s_, signaling the commencement of the globularization process [35,36]. Upon comparing the lamellar structures depicted in the SEM images of Figure 5c,d, it is apparent that the lamellar in the 950T-MF sample shows considerably less twisting and deformation when contrasted with those in the 900T-MF sample. The variation stems from the uniform distribution of the β phase when the lamellar acts as the matrix in the 950T-MF sample. Consequently, this uniform distribution leads to a more consistent deformation throughout the sample, reducing susceptibility to stress concentration areas, a characteristic observed in the 900T-MF sample. As depicted in Figure 5d, the lamellar in the 900T-MF sample exhibits greater distortion, making it challenging to discern the original lamellar structure. Additionally, the distribution of the β phase in the 900T-MF sample is more irregular when contrasted with that in the 950T-MF sample. This phenomenon arises from the configuration in the 900T-MF sample, where an equiaxed structure functions as the matrix during deformation. In this setup, a limited portion of the more deformable lamellar becomes the stress concentration region. As a result, the lamellar in the 900T-MF sample is more susceptible to fracturing in comparison to those in the 950T-MF sample.

### 3.3. Microstructure Evolution during Annealing

**Figure 8:**
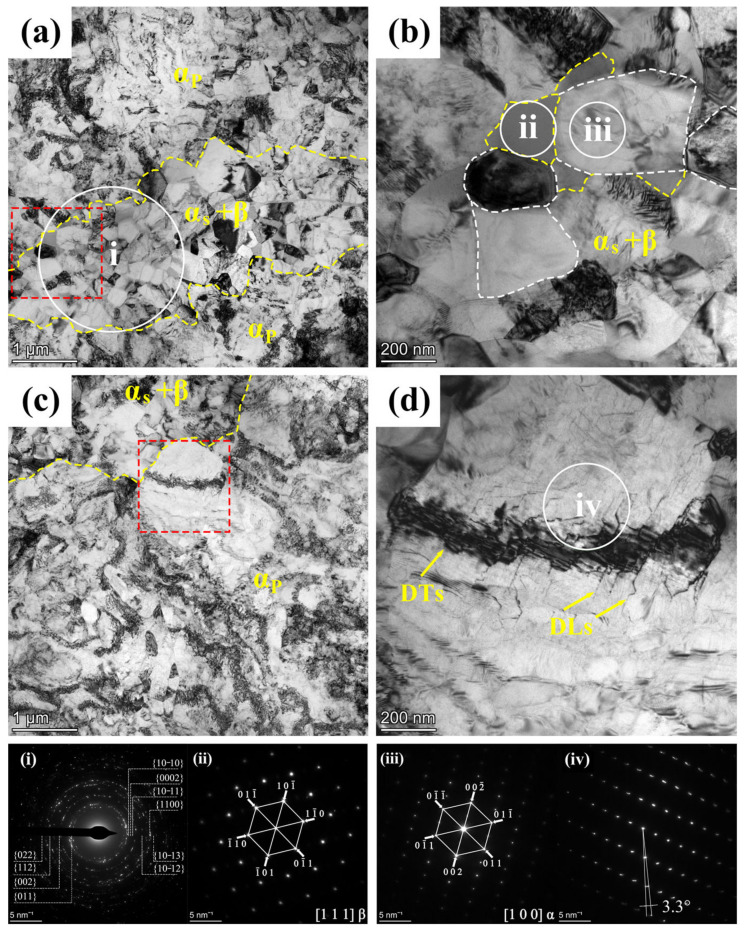
Bright-field TEM images of the 900T-MF-A sample: (**a**) Overview of α_p_ and α_s_+β regions microstructures; (**b**) α_s_ and β grain structures within the red-boxed region in (**a**); (**c**) Internal structure of α_p_ grains; (**d**) Dislocation structure within the α_p_ grain in the red-boxed region in (**c**,**i**–**iv**) (SAED patterns for marked areas).

Figure 8 presents the TEM bright field microstructure plot of the 900T-MF-A sample. After annealing in Figure 8a, the grain images demonstrate a significant decrease in the overall dislocation density compared to the 900T-MF samples. The reduction in dislocation density is particularly pronounced in the fine grains region, where the grains recover and grow to a certain extent. The SAED pattern of the i region in Figure 8a displays a ring-like diffraction pattern, signifying a polycrystalline nature. The density of diffraction rings in the region i of the 900T-MF sample, as seen in Figure 8a, is marginally lower than that observed in the corresponding region i of the 900T-MF sample in Figure 6a. This discrepancy can be attributed to the larger grain size in the 900T-MF-A sample compared to the 900T-MF sample. Furthermore, a reduction in dislocation density was noted in the α_p_ grain. However, complex DTs within the crystal, known as GNDs, are still observed. The α_s_ and β grains of the 900T-MF-A sample are visible in Figure 8b. Upon comparison with the 900T-MF sample shown in Figure 6b, it is evident that annealing increases the grain size, accompanied by a substantial reduction in dislocation density within the grains. In the fine grain region, the statistical analysis reveals that the average grain size of the 900T-MF-A sample is 177.4 ± 20 nm. This average grain size is 20.8% larger than that observed in the fine grains region of the 900T-MF samples.

Moreover, within the α_p_ region, a morphology resembling the striped structure seen in Figure 6c was identified, as depicted in Figure 8c. The SAED analysis indicated that region iv comprises an α phase with a zone axis of [001]. In Figure 8iv, the diffraction spots were not distinctly arranged in a lattice, and spot splitting was observed in the outer region, suggesting a misorientation angle of 3.3°. This observation indicates the presence of low-angle boundaries (LABs) in the circular regions. A similar structure was discussed by Liu [23] in a previous study, demonstrating that certain regions with DTs within the α_p_ grains transform into LABs. This structure is characterized by DLs emanating from the DTs, dividing grains into elongated or lens-shaped regions and forming LABs, representing an early stage in the twinning process.

**Figure 9:**
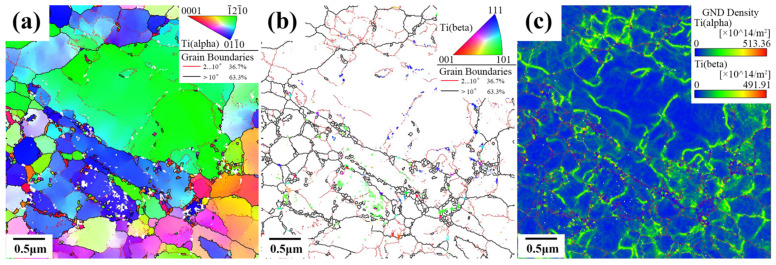
Microstructure analysis from TKD of the 900T-MF-A sample: (**a**) IPF and Grain Boundary map of α phase, (**b**) IPF and Grain Boundary map of β phase, (**c**) Kernel average misorientation distribution.

The TKD image post-annealing is depicted in Figure 9. The composition analysis reveals that the proportion of α phase is 98.1%, while the proportion of β phase is 1.9%. In Figure 9a, a polar diagram of the α phase is presented, allowing for the clear distinction between α_p_ grains and broken α_s_ grains. The orientation of adjacent α_s_ grains exhibits a discernible correlation, proving that these broken α_s_ grains originate from the same parent grain, specifically from the same α_s_ lamellar. In Figure 9b, a polar diagram of the β phase is presented, allowing for an intuitive observation of the distribution of the β phase in the lamellar structure after fragmentation. Notably, the β phase is predominantly distributed in the fine-grained region, and it is evident that these particles are formed after the fragmentation of the lamellar phase within the lamellar structure. Interestingly, a few nanoscale β grains are also observed within the α_p_ grains distributed along the LABs. The formation of these β grains may be attributed to phase transformation induced by deformation. Liu et al. [37] similarly discovered that the Ti-6Al-4V alloy transitions from α-Ti with partial hcp structure to β-Ti with bcc structure after SPD. The proposed mechanism for this transformation involves the gliding of Shockley partial dislocations.

The Kernel Average Misorientation (KAM) map, as depicted in Figure 9c, is utilized to characterize the density of GNDs. In this map, different colors represent varying dislocation densities induced by different degrees of deformation. In this context, the blue region corresponds to the lowest dislocation density, indicating minor deformation. In contrast, the green region is represented as an area with higher dislocation density resulting from more significant deformation. The KAM map in Figure 9c unveils an intriguing observation: the GNDs in the fine-grained region are predominantly distributed at grain boundaries, with a minimal presence within the grains. Within the α_p_ grain, numerous GNDs are observed, distributed along the LABs. This observation suggests that, in the fine-grained region, there is essentially no substructure within the grains. In contrast, within the α_p_ grains, the substructure and GNDs are still present.

### 3.4. X-ray Diffraction Analysis

**Figure 10:**
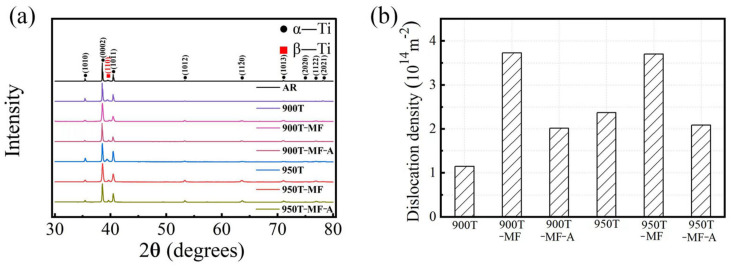
XRD profiles of Ti-6Al-4V alloy. (**a**) a stacked view and (**b**) bar graph of dislocation density calculated from XRD data.

Figure 10 presents the XRD pattern and analytical data of the sample. The XRD image of the samples is displayed in Figure 10a. It has been verified that both the α and β phases persist and remain after successive heat treatment, MF, and annealing. Upon comparing the (10$\bar{1}$1) peaks in Figure 10a, it is evident that the (10$\bar{1}$1) peaks of the 900T and 950T samples shift to the right compared to the AR sample. MF can also induce peak shifts, a phenomenon attributed to lattice distortion resulting from residual stress.

Using Hall’s method, Formula (1) establishes a connection between the grain size and lattice strain based on the 2*θ* angle and FWHM [38]:

(1) $$\frac{{\left(\delta 2\theta \right)}^{2}}{{tan}^{2}\theta_{0}}=25\langle e^{2}\rangle +\frac{\lambda }{d}\left(\frac{\delta 2\theta }{\tan\theta_{0}\sin\theta_{0}}\right)$$

Replace the 2*θ* and FWHM values obtained from the main peak measured by XRD into formula (1) and conduct linear fitting on the outcomes. Subsequently, calculate the lattice strain and grain size. Ultimately, compute the dislocation density using Formula (2) [39]:

(2) $$\rho =2\sqrt{3}{\langle e^{2}\rangle }^{1/2}/(db)$$

*b* represents the Burgers vector, with a value of 0.295 nm. The resulting dislocation density is depicted in Figure 10b. It is evident that the dislocation density for the 900T-MF and 950T-MF samples is relatively high, around 3.7 × 10^14^ m^−2^. Following annealing, the dislocation density for both samples decreases to approximately 2.1 × 10^14^ m^−2^.

### 3.5. Deformation Mechanism

**Figure 11:**
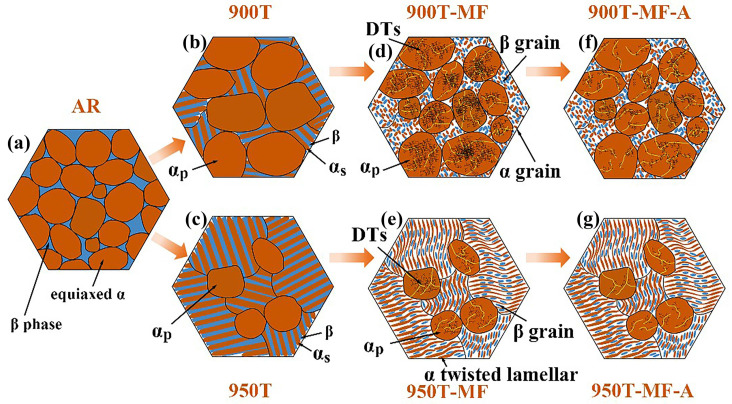
Diagrammatic representation depicting the heat treatment and deformation modes of the Ti-6Al-4V alloy: microstructure of (**a**) AR sample, (**b**) 900T sample, (**c**) 950T sample; morphology of (**d**) α_p_ grain and lamellar structure of 900T-MF sample; (**e**) α_p_ grain and lamellar structure of 950T-MF sample; (**f**) grain and dislocation of 900T-MF-A sample; (**g**) grain and dislocation of 950T-MF-A sample.

Utilizing the findings from the experiments, a comprehensive diagram illustrating the deformation process of the Ti-6Al-4V alloy is depicted in Figure 11. As the temperature of the heat treatment progressively approaches T_β_, the β phase, initially concentrated at grain boundaries, serves as nucleation sites and undergoes gradual growth. Subsequently, air cooling is applied to facilitate the precipitation of the α_s_+β lamellar structure. With the heat treatment temperature rising from 900 °C to 950 °C, there is a decrease in the number of α_p_ grains, accompanied by a gradual increase in the ratio of α_s_+β lamellar.

During the MF process, the lamellar microstructure experiences distortion and fragmentation. When deformation samples containing a high proportion of lamellar microstructures, as observed in samples 950T-MF, the grains from various lamellar microstructures work in coordination, leading to a relatively uniform overall deformation of the sample. With the gradual increase in the content of α_p_ grains, the grains of the lamellar microstructure become segmented into discontinuous regions. This leads to local stress concentration in the area of the lamellar microstructure during plastic deformation. In the deformation process, samples featuring a higher content of α_p_ grains, as exemplified by sample 900T-MF, show more pronounced fragmentation in the lamellar microstructure area. In such cases, particles from the α_s_ lamellar and β lamellar are uniformly mixed. Deformation of the α_p_ grain results in the formation of substructures like DTs and LABs. In the 900T sample, the α_p_ grains undergo a higher degree of deformation than the 950T sample, leading to DTs and subgrains within the grains. The cumulative presence of these substructures within the grains eventually contributes to the fragmentation of certain α_p_ grains. Following the annealing process, the dislocation density within the grains of the 900T-MF and 950T-MF samples decreased. UFG structures exhibit a limited extent of recovery and growth, with no GNDs present inside the grains. However, GNDs persist within the α_p_ grains at the LABs. The α_p_ grains function as reservoirs for storing dislocations during the deformation process, and the varying proportions of α_p_ grains ultimately result in differences in the ability of the 900T-MF and 950T-MF samples to store dislocations.

### 3.6. Mechanical Properties

#### 3.6.1. Tensile Testing

**Figure 12:**
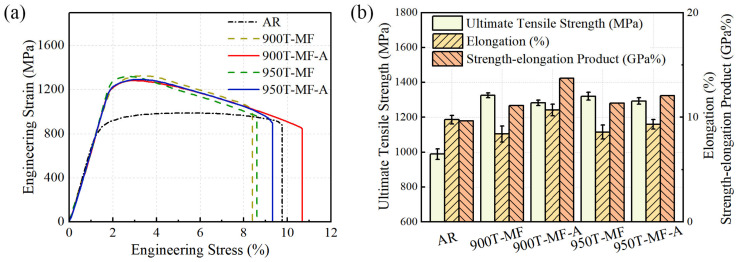
Tensile test data of Ti-6Al-4V alloy: (**a**) Engineering stress vs strain curve; (**b**) Bar graph of UTS, TE, and strength–elongation product.

The tensile curves for the five different samples are depicted in Figure 12a. The UTS and TE at failure for the 900T-MF and 950T-MF samples are similar. However, the UTS of the 900T-MF-A sample remains notably high, surpassing 1280 MPa, in contrast to the UTS of the 900T-MF sample, which is at 1324 MPa. The total elongation of the 900T-MF-A sample reached 10.7%, marking a 27% increase compared to the 900T-MF sample. In contrast, the total elongation growth of the 950T-MF-A sample is not significant, owing to the distinct effects of annealing on the two samples. In the case of the 900T-MF sample with a higher content of α_p_ grains, annealing eliminates dislocations within the α_p_ grains. This process creates more available areas for storing dislocations.

In the 950T sample, characterized by a higher lamellar structure, many fractured grains formed after the MF process. This resulted in a significant increase in the grain boundary area, impeding the movement and diffusion of dislocations, thereby impacting the material’s ductility. The subsequent annealing process resulted in only slight growth of small grains in the 950T-MF-A sample, and there was no significant change in the number of grain boundaries. Therefore, the effect of annealing on total elongation in the case of the 950T-MF-A sample was not significant.

The present observations diverge somewhat from the data presented by Zherebtsov [18]. According to their report, the elongation of the bimodal UFG Ti-6Al-4V alloy, obtained through the MF of the Ti-6Al-4V alloy featuring equiaxed α grains and a lamellar structure, can directly achieve a remarkably high level. Nevertheless, in the current study, it has been noted that the elongation of the bimodal microstructure Ti-6Al-4V alloys decreases compared to the base material. However, this reduction is considerably less pronounced than in materials obtained through traditional SPD techniques. It is noteworthy that after annealing, the total elongation of the material surpasses even that of the base material. This situation can be explained from two perspectives. Firstly, within this bimodal structure, two types of structures give rise to two distinct deformation modes during tensile deformation: α_p_ grains, characterized by multiple sliding, and areas with a very fine deformation relief typical of a fine-grained microstructure [18]. The α_p_ grains, lacking well-developed substructures and high-angle boundaries, can function as sites for dislocation storage. Many dislocations accumulated within the α_p_ grain during the plastic deformation stage are removed through annealing. This process enhances the ability of the α_p_ grain to accumulate dislocations and consequently improve plasticity. Moreover, the strength–elongation product was calculated, as illustrated in Figure 12b. Strength-elongation product is an indicator used to characterize the comprehensive properties of metal materials, which represents the product of the UTS and TE of metal materials. Following the annealing treatment, there has been a notable improvement in the strength–elongation product, with the 900T-MF-A sample exhibiting a higher value than that of the base material. This suggests that bimodal UFG materials exhibit a superior degree of strength–elongation matching compared to completely UFG materials.

#### 3.6.2. Tensile Fracture Morphology

**Figure 13:**
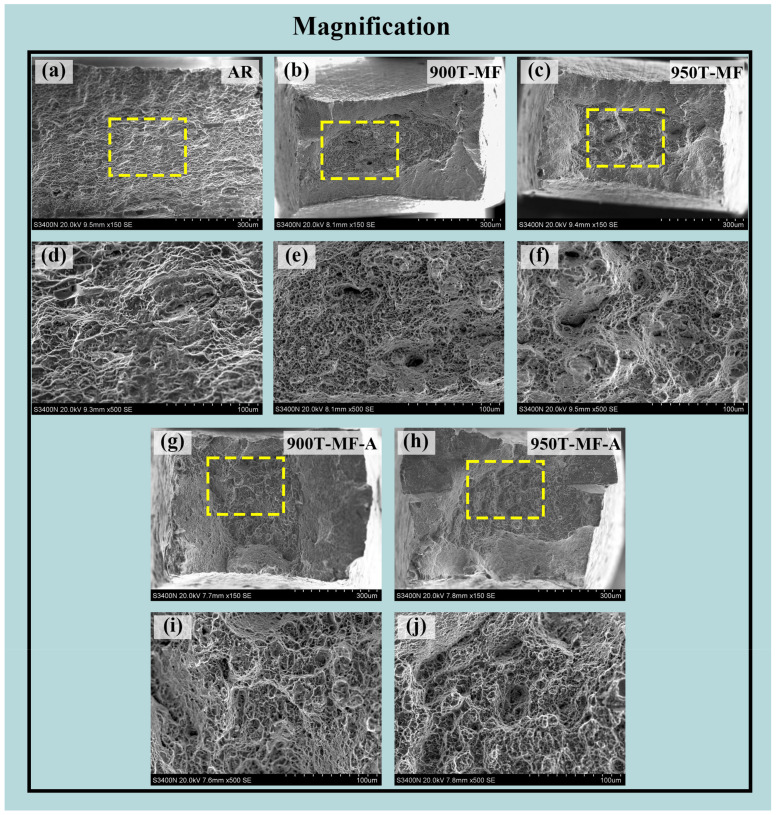
SEM images of fracture surfaces of different samples: (**a**,**d**) AR-Ti6Al4V; (**b**,**e**) 900T-MF sample; (**c**,**f**) 950T-MF sample; (**g**,**i**) 900T-MF-A sample; (**h**,**j**) 950T-MF-A sample.

The representative fracture surfaces of the five samples are depicted in Figure 13, with the fracture characteristics corresponding to the changes in the plasticity of the samples. In Figure 13a, the overall morphology of the fracture surface of the AR sample is presented, revealing an absence of a shear lip on the fracture surface. In Figure 13d, it is evident that the overall size of the dimples is larger, and the morphology appears more uniform compared to other samples. This characteristic is attributed to the equiaxed α grains within the AR sample structure. Some large ductile dimples exhibit partial tearing edges, and the overall sample displays ductile fracture characteristics. For the 900T-MF sample and 950T-MF sample (Figure 13b,c), the necking zone, characterized by shear lips on the periphery, exhibits a cup-shaped structure, indicative of a ductile fracture mode at room temperature. The central fiber area of the fracture surface represents the plane strain portion of the unstable propagation of tensile cracks. Numerous deep equiaxed dimples are distributed in the fiber area of the fracture surface (Figure 13e,f), which is attributed to the process of pore nucleation, growth, and subsequent aggregation during tensile loading [40]. In Figure 13e,f, it is evident that the morphology of the dimples is largely consistent between the two. The presence of numerous deep dimples is a characteristic feature of plastic materials. However, in Figure 13f, a small number of large-sized pits exhibiting quasi-cleavage fracture characteristics are observed. Following annealing, the average size of the fracture dimples in the two samples (Figure 13g,h) increases. It is noticeable that the central fiber area of the annealed samples is larger than that of the MF samples, with the central fiber area of the 900T-MF-A sample also being larger than that of the 950T-MF-A sample. A larger fiber zone may suggest the material has experienced substantial plastic deformation during fracture.

#### 3.6.3. Microhardness

**Figure 14:**
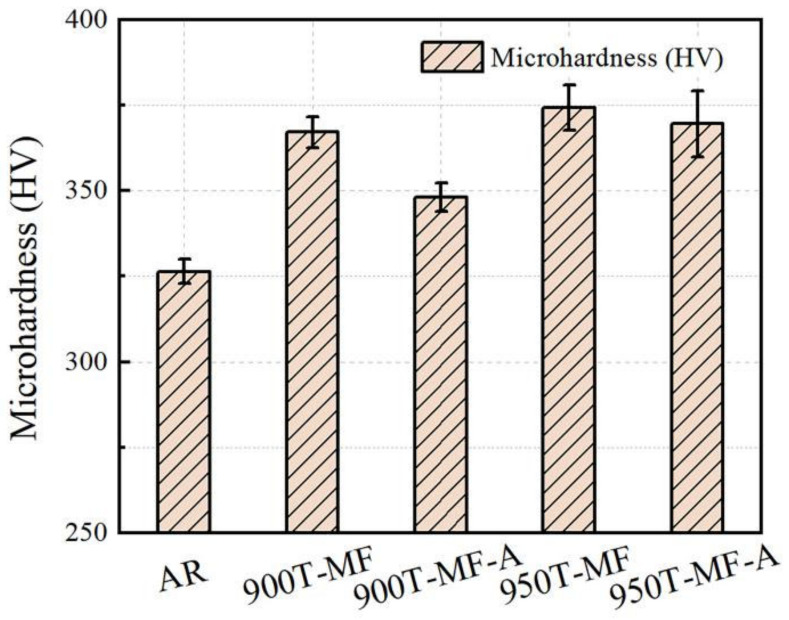
Vickers microhardness values.

The microhardness comparison of the AR sample and two types of samples after forging and annealing is illustrated in Figure 14. The microhardness of the 900T-MF and 950T-MF samples reached 367.1 HV and 374.2 HV, respectively. The disparity in hardness between the two samples arises from the variance in microstructure.

The 950T sample exhibits a larger proportion of lamellar structure, and after MF, more fine grain areas are formed, contributing to higher hardness compared to the 900T-MF sample. After annealing, the hardness of both samples decreased; however, the hardness of the 900T-MF-A decreased more significantly to 348.0 HV. This difference is attributed to variations in the organizational structure. The 950T-MF sample possesses more fine-grained areas and a more pronounced grain boundary-strengthening effect. Even after annealing, grain boundaries persist, resulting in minimal impact on the hardness of the material.

## 4. Summary and Conclusions

There are distinct deformation mechanisms between equiaxed and lamellar structures in the Ti-6Al-4V titanium alloy. This study investigates the deformation mechanisms and differences in mechanical properties among various proportions of lamellar and equiaxed structures during MF and annealing. The key conclusions are as follows:

1. Holding the material at 900 °C for 1 h, followed by air cooling, results in a microstructure where α_p_ grain constitutes 58.7%, and the average grain size measures 16.7 ± 2 μm. The thickness of the α_s_ lamellar and β lamellar structures is reported as 0.4 ± 0.2 µm and 0.08 ± 0.02 µm, respectively. Following a heat treatment holding at 950 °C for 1 h and subsequent air cooling, the resulting sample exhibits an α_p_ grain proportion of 12.8%, accompanied by an average grain size of 5.1 ± 0.8 μm. The thickness of the α_s_ lamellar and β lamellar structures is slightly thicker.
2. The α_p_ grain undergoes significant deformation during the MF process, and its morphology remains equal. A substantial amount of DTs and LABs are generated inside the α_p_ grains, and some α_p_ grains are broken into smaller grains. During the MF process, the α_s_+β lamellar structure of the 900T-MF sample undergoes complete globularization, forming a UFG region with an average grain size of 146.9 nm. The average grain size of α_p_ grains decreases to 7.3 ± 0.6 μm. In contrast, the 950T-MF sample initially breaks down the β lamellar structure, while the α_s_ lamellar undergoes distortion but does not completely globularize. The average grain size of the α_p_ grain remains unchanged at 4.7 ± 1.2 μm.
3. Following annealing, the grains in the fine-grained region of the sample increased to a certain extent, and the overall dislocation density decreased. Some GNDs were retained in the α_p_ grains, and the average grain size in the UFG region of the 900T-MF-A sample increased to 177.4 nm.
4. The 900T-MF-A sample demonstrated the highest strength–elongation product, achieving a UTS of 1280 MPa, TE of 10.7%, and a 6.7% increase in microhardness. The fracture surfaces of both MF and annealed samples exhibit characteristics dominated by ductile fracture.

## Figures and Tables

**Table 1:** Elemental composition of as-received Ti-6Al-4V alloy.

Element	Al	V	Fe	C	N	H	O	Ti
% wt.	5.9	4.2	0.2	0.15	0.05	0.015	0.15	Bal.

## Data Availability

All data included in this study are available upon request by contact with the corresponding author.
